# Genome-wide identification of the GATA transcription factor family and their expression patterns under temperature and salt stress in *Aspergillus oryzae*

**DOI:** 10.1186/s13568-021-01212-w

**Published:** 2021-04-19

**Authors:** Chunmiao Jiang, Gongbo Lv, Jinxin Ge, Bin He, Zhe Zhang, Zhihong Hu, Bin Zeng

**Affiliations:** Jiangxi Key Laboratory of Bioprocess Engineering and Co-Innovation Center for In-Vitro Diagnostic Reagents and Devices of Jiangxi Province, College of Life Sciences, Jiangxi Science and Technology Normal University, Nanchang, 330013 China

**Keywords:** *Aspergillus oryzae*, GATA transcription factors, Temperature stress, Salt stress, Gene expression

## Abstract

GATA transcription factors (TFs) are involved in the regulation of growth processes and various environmental stresses. Although GATA TFs involved in abiotic stress in plants and some fungi have been analyzed, information regarding GATA TFs in *Aspergillus* *oryzae* is extremely poor. In this study, we identified and functionally characterized seven GATA proteins from *A.* *oryzae 3.042* genome, including a novel AoSnf5 GATA TF with 20-residue between the Cys-X_2_-Cys motifs which was found in *Aspergillus* GATA TFs for the first time. Phylogenetic analysis indicated that these seven *A*. *oryzae* GATA TFs could be classified into six subgroups. Analysis of conserved motifs demonstrated that *Aspergillus* GATA TFs with similar motif compositions clustered in one subgroup, suggesting that they might possess similar genetic functions, further confirming the accuracy of the phylogenetic relationship. Furthermore, the expression patterns of seven *A.* *oryzae* GATA TFs under temperature and salt stresses indicated that *A. oryzae* GATA TFs were mainly responsive to high temperature and high salt stress. The protein–protein interaction network of *A.* *oryzae* GATA TFs revealed certain potentially interacting proteins. The comprehensive analysis of *A. oryzae* GATA TFs will be beneficial for understanding their biological function and evolutionary features and provide an important starting point to further understand the role of GATA TFs in the regulation of distinct environmental conditions in *A.* *oryzae*.

**Supplementary Information:**

The online version contains supplementary material available at 10.1186/s13568-021-01212-w.

## Introduction

GATA transcription factors (TFs) constitute a family proteins that is characterized by the presence of one or two highly conserved type-IV zinc fingers (Cys-X_2_-Cys-X_17-20_-Cys-X_2_-Cys) and a DNA-binding domain that recognizes the conserved (A/C/T)-G-A-T-A- (A/G) sequence in the promoter sequence of target genes (Scazzocchio 2000; Lowry and Atchley 2000). In fungi, GATA TFs are mainly involved in nitrogen regulation and light responses, regulation of sexual and/or asexual reproduction, and secondary metabolism. GATA TFs *AreB* and *AreA* are not only involved in nitrogen and carbon metabolism, but also in the control of several complex cellular processes such as transport and secondary metabolism (SM) (Pfannmüller et al. 2017; Chudzicka-Ormaniec et al. 2019). *SreA* is involved in the regulation of siderophore biosynthesis and iron uptake (Oberegger et al. 2010; Schrettl et al. 2008), and *NsdD* regulates sexual and/or asexual reproduction and the production of SMs (Lee et al. 2014, 2016; Niehaus et al. 2017). Furthermore, few fungal GATA TFs also play important roles in response to abiotic stresses. *Alternaria alternata SreA* is related to the maintenance of cell wall integrity (Chung et al. 2020), while *Blastomyces dermatitidis SreB* strongly expresses and contributes to filamentous growth at 22 ℃ via lipid metabolism (Marty et al. 2015). Additionally, *GLN3* and *GAT1* have been shown to be involved in salt tolerance in *Saccharomyces cerevisiae* (Crespo et al. 2001). However, reports regarding the function of GATA TFs filamentous fungi in response to abiotic stress factors are limited.

*Aspergillus oryzae* is an important filamentous fungus, which is widely used in East Asian traditional fermented food products (Kitamoto 2015). During fermentation, *A.* *oryzae* is exposed to various environmental stress factors. Temperature is the most important environmental factor affecting the growth and activity of microorganisms and can directly affect the activity of enzymes involved in substrate digestion during fermentation process (Chen et al. 2011; Bechman et al. 2012). In addition, high sodium chloride concentration, which inhibits the growth of spoilage bacteria in soy sauce mash, also affects the growth of *A.* *oryzae* (Wang et al. 2013; Fernandes et al. 2018). Therefore, the ability of *A.* *oryzae* to adapt to different temperatures and high salt concentrations have attracted attention, although the molecular mechanisms underlying their response to these stress factors still remain unclear. Previous studies have demonstrated that GATA TFs are mainly involved in regulation of various temperature and salt stress stimuli in few fungi (Scazzocchio 2000; Crespo 2001; Marty et al. 2015). Although the Fungal Transcription Factor Database (FTFD) and Kobayashi et al. have publicized six *A. oryzae* GATA TFs, which are involved in nitrogen regulation, light responses, regulation of sexual and/or asexual reproduction, and SM (Kobayashi et al. 2007), studies regarding a comprehensive analysis of *A. oryzae* 3.042 GATA TFs are lacking. Therefore, the aim of this study was to analyze the structural characteristics, evolutionary features, conserved motifs, and expression patterns of *A.* *oryzae* GATA TFs under temperature and salt stress. Furthermore, the expression patterns and the results of protein–protein interaction (PPI) can establish a good foundation for further studies on the function and the mechanism of *A.* *oryzae* GATA TFs in abiotic stress responses.

## Materials and methods

### Identification of *A. oryzae* GATA TFs

The *A.* *oryzae* *3.042* genome was downloaded from NCBI database (https://www.ncbi.nlm.nih.gov/genome/?term=Aspergillus+oryzae). The BLASTP program, with a threshold e-value of 1e-10, was used to predict GATA TFs in the *A.* *oryzae 3.042* genome, using gene sequences from *Aspergillus* as query sequences. All potential *A.* *oryzae* GATA TFs were identified using HMMER3.1 and were predicted if they contained ZnF-GATA domains (PF00320). The sequences that generated hits with GATA-type zinc finger genes encoding GATA zinc-finger domains (PF00320) were considered as GATA TFs. CDD and PFAM databases were used to validate all the potential *A.* *oryzae* GATA TFs. Finally, seven query sequence IDs of Ao3042_00752, Ao3042_04581, Ao3042_01136, Ao3042_04436, Ao3042_04150, Ao3042_05944, and Ao3042_05500 contain ZnF-GATA domains.

To determine the chromosomal locations of the seven identified *A. oryzae* GATA TFs, locus coordinates were downloaded from the *A.* *oryzae* RIB40 genomics database. The distribution of seven *A.* *oryzae* TFs on the chromosomes was drawn using MG2C (mg2c.iask.in/mg2c_v2.0/) and visualized using MapChart 2.2 (Voorrips 2002).

### Multi sequence alignment and phylogenetic analysis

ClustalW was used to align *A.* *oryzae* GATA TF proteins. The protein sequences of known GATA TFs in all other *Aspergillus* were downloaded from fungal transcription factor databases (FTFD, http://ftfd.snu.ac.kr/index.php?a=view). The sequences of GATA TFs in *A.* *oryzae* and other *Aspergillus* species were also aligned using ClustalW to analyze the phylogenetic relationships of all *Aspergillus* GATA TFs. A neighbor-joining (NJ) tree was constructed based on alignment results in MEGA6.0 with bootstrap replications of 1000. The sequence IDs of GATA TFs used to construct the phylogenetic NJ tree is shown in Additional file 1: Table S1.

### Motif analysis of *A. oryzae *and other *Aspergillus* GATA transcription factors

MEME was used to predict and analyze motifs of *A.* *oryzae* GATA proteins, which were visualized using TBtools (Chen et al. 2011). The parameters were set to zero or one of a contributing motif site per sequence, and the numbers of motifs chosen was five; motif widths were set to 6 and 50 (Wu et al. 2016). The other parameters were set to default values. Each motif was individually checked so that only motifs with e-value of < 1e-10 were retained for motif detection in *A.* *oryzae* GATA proteins.

### Effects of temperature and salinity treatment on *A. oryzae* growth

*A. oryzae* 3.042 (CICC 40,092), the main fermentation strain used in industry, was selected to test the growth of *A.* *oryzae* under temperature and salt stress. *A.* *oryzae* conidia were inoculated in fresh potato dextrose agar (PDA) medium and cultured at 22, 25, 30, 35 and 42 °C for 72 h to investigate the effects of temperature; the optimum growth temperature of *A.* *oryzae*, 30 °C, was used as the control temperature. PDA media with final salt concentration of 5.0, 10.0, 12.5 and 15.0 g/100 mL NaCl were prepared to assess the effects of salinity stress on *A. oryzae*. Medium without salt was used as the control medium. Two microliters of freshly prepared *A.* *oryzae* suspension containing 1 × 10^7^ conidia were inoculated on the medium to analyze phenotypes. To determine the effect of these two abiotic stresses on fungal viability, 100 µL of 1 × 10^7^ conidia suspension was inoculated per plate covered with cellophane (Solarbio, Beijing, China); the fungal mycelia were collected after 72 h of incubation. The fungal mycelia were then dried overnight, and the dry biomass was tested. Material for RNA extraction was also collected simultaneously. The experiments were performed in triplicate.

### Quantitative real-time polymerase chain reaction (qRT-PCR) for analyzing the expression of *A. oryzae* GATA TFs in response to temperature and salinity stress

Total RNA was extracted using an Omega plant RNA kit (Omega Bio-Tek, Georgia, USA) according to the manufacturer’s instructions. One microgram of RNA was reverse-transcribed into cDNA using PrimeScript™ RT reagent with the gDNA Eraser kit (TaKaRa, Dalian, China). *A. oryzae* GATA TF primers were designed using the Primer-BLAST tool (https://www.ncbi.nlm.nih.gov/tools/primer-blast) (Additional file 2: Table S2). Gene expression levels were determined by perfoming qRT-PCR on a Bio-rad CFX96 Touch instrument (Bio-Rad, USA) using TB Premix Ex Taq II (TaKaRa) according to the manufacturer's instructions. Data were analyzed using Bio-rad CFX96 software and the ${2^- }^{\vartriangle \vartriangle {C_T}}$ method (Livak and Schmittgen 2001). The histone H1 gene was used as the reference gene in qRT-PCR analysis.

### Construction of protein–protein interaction network

Protein–protein interaction (PPI) data were obtained from the online database of STRING (https://string-db.org/), which is an open source software for predicting and visualizing complex networks. These interactions were derived from literature regarding experimental validation of physical interactions and enzymatic reactions associated with signal transduction pathways. The PPI networks were visualized in biological graph-visualization tool Cytoscape with the nodes representing proteins/genes (Pathan et al. 2015).

## Results

### Characteristics of *A. oryzae* GATA TFs

BLASTP analysis was used to check predicted the GATA TFs from the *A.* *oryzae* *3.042* genome. All potential *A.* *oryzae* GATA proteins were used to identify ZnF_GATA domains (PF00320) using HMMER3.1. In total, seven *A. oryzae* GATA TFs were identified, and were named *AoAreA*, *AoAreB*, *AoLreA*, *AoLreB*, *AoNsdD*, *AoSnf5* and *AoSreA* corresponding to the names of fungal orthologs (Table 1). The lengths of the *A.* *oryzae* GATA TFs ranged from 313 (AoAreB) to 867 (AoAreA) amino acid. The details of these *A. oryzae* GATA TFs, such as ZnF_GATA motif type, number of ZnF_GATA domains, sizes of the deduced peptides, and their homologous gene IDs, are listed in Table 1.

**Table 1:** The characteristics of *A. oryzae* GATA TFs

Name	Protein ID	Peptide (aa)	ZnF_GATA Motif type	Number domain of ZnF_GATA	Homologous ID	Extra domain
AoSreA	EIT82081.1	567	Cys-X_2_-Cys-X_17_-Cys- X_2_-Cys	2	KOC08900.1	TFIIB zinc-binding
AoAreB	EIT79032.1	313	Cys-X_2_-Cys-X_17_-Cys- X_2_-Cys	1	XP_002379623.1	TFIIB zinc-binding
AoAreA	EIT72728.1	867	Cys-X_2_-Cys-X_17_-Cys- X_2_-Cys	1	RAQ50831.1	AreA_N
AoLreB	EIT79273.1	496	Cys-X_2_-Cys-X_18_-Cys- X_2_-Cys	1	RAQ50386.1	PAS
AoNsdD	EIT79449.1	504	Cys-X_2_-Cys-X_18_-Cys- X_2_-Cys	1	KOC07076.1	-
AoLreA	EIT77832.1	283	Cys-X_2_-Cys-X_18_-Cys- X_2_-Cys	1	XP_002384232.1	PAS
AoSnf5	EIT78280.1	570	Cys-X_2_-Cys-X_20_-Cys- X_2_-Cys	1	XP_022385751.1	SNF5/INI1

**Fig. 1:**
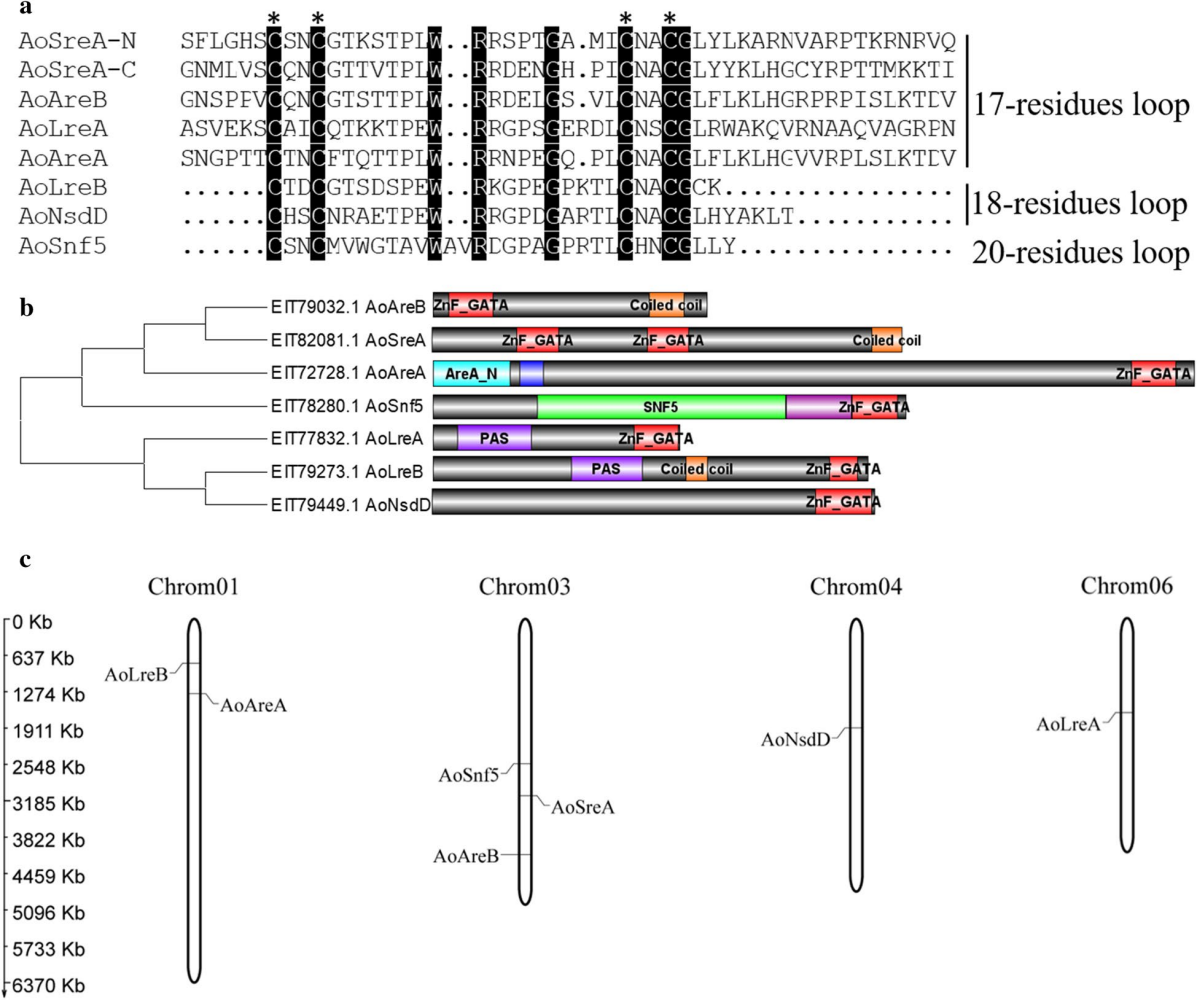
Alignment of conserved domain, prediction of functional domains, and chromosomal location of *A. oryzae* GATA TFs. **a** Alignment of the DNA interacting domain of *A. oryzae* GATA TFs. Cysteines from the Cys-X_2_-Cys*-*X_17/18/20_-Cys-X_2_-Cys domain are indicated by an asterisk above the sequence alignment. The 17, 18, and 20 numbers indicate the amino acid residues between Cys-X_2_-Cys. **b** Seven *A. oryzae* GATA proteins were aligned and clustered using MEGA6.0, and their ZnF_GATA domains are shown in red beside the neighbor-joining tree. **c** The distribution of *A.* *oryzae* GATA TFs on chromosomes. The vertical columns represent chromosomes; gene names are shown at the side of chromosomes

The GATA DNA binding domain is a conserved type-IV zinc-finger motif containing the Cys-X_2_-Cys-X_17-20_-Cys-X_2_-Cys motif. The zinc-finger motifs of Cys-X_2_-Cys -X_17-20_-Cys -X_2_-Cys differed among the seven* A. oryzae* GATA proteins. Six* A. oryzae* GATA domains contained the Cys-X_2_-Cys-X_17/ 18_-Cys-X_2_-Cys motif as reported in other fungi, while 20 residues were present in the zinc-finger loop of AoSnf5 between the Cys-X_2_-Cys motifs, which has rarely been found in fungi (Teakle and Gilmartin 1998; Scazzocchio 2000) (Table 1; Fig. 1a). Interestingly, AoSreA harbored two highly conserved type-IV zinc-finger motifs with Cys-X_2_-Cys-X_17_-Cys-X_2_-Cys (Table 1; Fig. 1a), which usually occur in animals (Patient and Mcghee 2002). Apart from the ZnF_GATA domain, additional domains such as TFIIB zinc-binding domain, AreA-N, SNF5/INI1, and PAS were also characterized (Table 1; Fig. 1b). Previous studies have demonstrated that the PAS domain mainly functions in sensing environmental or physiological signals including oxidative and heat stress (Nan et al. 2011; Corrada et al . 2016). Therefore, extra domains present in* A. oryzae* GATA may also play the same role in diverse environmental stresses and may facilitate the functional analysis of* A. oryzae* GATA TFs.

In addition, the chromosomal location of *A.* *oryzae* GATA TFs revealed their random distribution in the *A. oryzae* genome. Here, the seven GATA TFs of *A.* *oryzae* 3.042 were mapped to the first complete genome of *A.* *oryzae* strain RIB40. The chromosomal distribution of *A.* *oryzae* GATA TFs was visualized using the MapChart program. The seven *A.* *oryzae* GATA TFs were randomly distributed on chromosomes 1, 3, 4, and 6 (Fig. 1c). Interestingly, AoAreB, AoSreA, and AoSnf5 clustered into the same subgroup in the NJ tree (Fig. 1b) and were distributed on the same chromosome, which indicates a close evolutionary relationship among them. The chromosomal location of *A.* *oryzae* GATA TFs may assist in determining the exact sequence of events.

### Phylogenetic analysis of the *Aspergillus* GATA TFs

**Fig.2:**
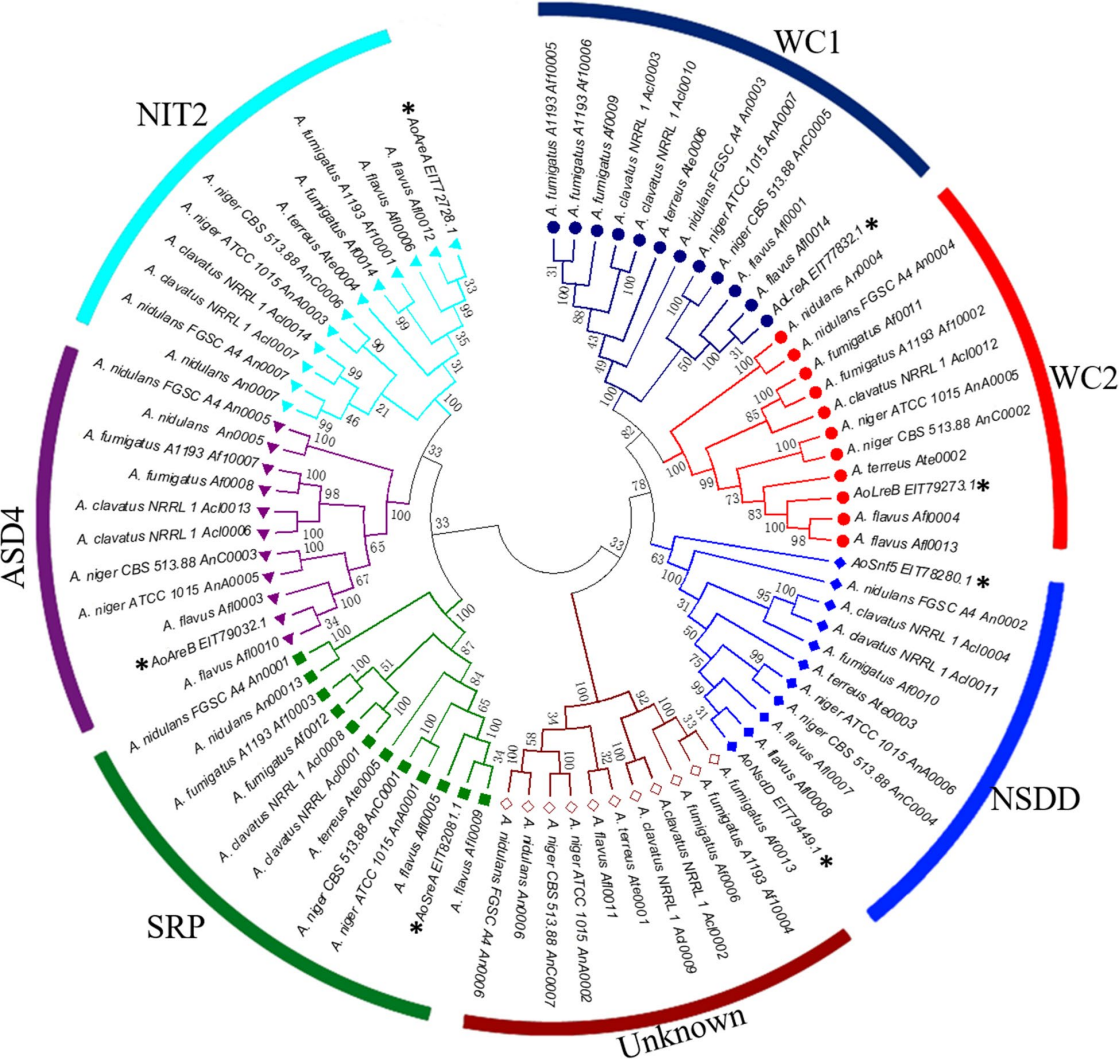
Phylogenetic analysis of *A. oryzae* and other *Aspergillus* TFs*.* GATA protein sequences were aligned using ClustalW in MEGA6.0 software using default parameters. The consensus NJ tree represents 1000 bootstrap replications. Bootstrap values are displayed as nodes. The protein sequences of *Aspergillus* GATA TFs were downloaded from FTFD. The *Aspergillus* GATA TFs are classified into seven subgroups in the NJ tree, including one group with unknown function. Seven *A. oryzae* GATA TFs are scattered in six known subgroups, and the novel AoSnf5 also clustered in the NSDD subgroups together with AoNsdD

A NJ_tree was constructed using MEGA6.0 for multiple sequence alignment of all *Aspergillus* GATA TFs with 1000 bootstrap replications to analyze phylogenetic relationships between the *A.* *oryzae* GATA TFs and other *Aspergillus* GATA TFs with ZnF_GATA domains. All the *Aspergillus* GATA TFs were divided into seven subgroups in the NJ tree based on the number of ZnF_GATA domains and zinc finger motifs of GATA domain sequences with other *Aspergillus* GATA TFs from FTFD, including six known subgroups of WC1, WC2, NSDD, SRP, ASD4, NIT2 and one unknown function subgroup (Fig. 2). Seven *A.* *oryzae* GATA TFs were scattered in the six subgroups with other *Aspergillus* GATA TFs, functions of which have been reported, while the novel AoSnf5 GATA TF also clustered in the NSDD subgroups together with AoNsdD. The function of the different GATA subgroups vary. For example, the GATA TFs of WC1 and WC2 subgroups are mainly involved in the regulation of blue- and red-light responses (Purschwitz et al. 2008; Purschwitz et al. 2013). Nitrogen regulation is regulated by the process of nitrogen catabolite repression which controls gene expression via GATA TFs of the NIT2 and ASD4 subgroup in yeasts and filamentous fungi (Pfannmüller et al. 2017; Pomraning et al. 2017; Michielse et al. 2014). Therefore, the AoLreA, AoLreB, AoAreA, and AoAreB divided into WC1, WC2, NIT2, and ASD4 subgroups, respectively, might also be involved in light responses or nitrogen regulation as reported. In addition, *NsdD* has been shown not only to affect sexual and asexual reproduction, but also secondary metabolism in *Aspergillus* (Lee et al. 2014, 2016), which could help in determining the function of *AoNsdD* and *Aosnf5* assigned to the NSDD subgroup.

### Analysis of conserved motifs in *A. oryzae* GATA TFs

**Fig.3:**
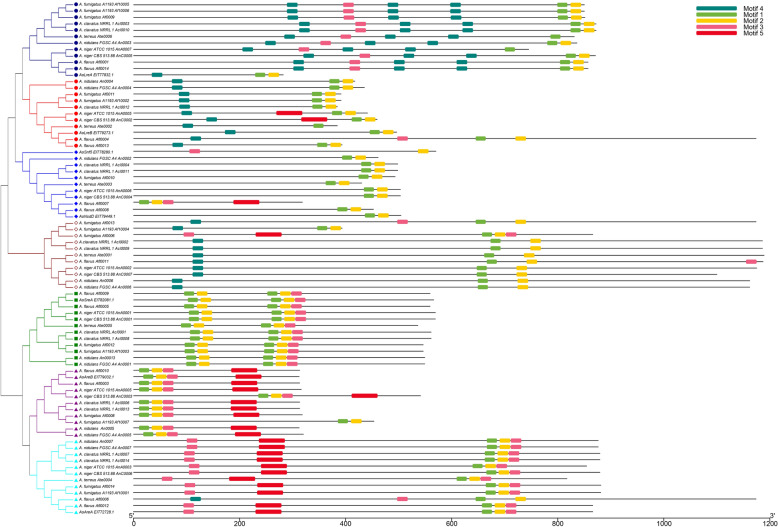
The conserved motif arrangement of *A. oryzae* and other *Aspergillus* GATA TFs based on their phylogenetic relationships. A NJ tree was predicted from the amino acid sequences of GATA TFs using ClustalW and MEGA6.0 with 1, 000 bootstrap replications. The conserved motifs in the GATA TFs were identified using MEME. In total, five conserved motifs were identified and are shown in different colors

To obtain insights regarding the diversity of motif compositions in *A.* *oryzae* GATA TFs, the conserved motifs in the *A.* *oryzae* and other *Aspergillus* GATA TFs were predicted using the MEME4.11.4 online software. In total, five conserved motifs were identified. The relative locations of these motifs within the protein are shown in Fig. 3. The identified consensus sequence of the five motifs is shown in Additional file 4: Figure S1. A typical zinc-finger structure composed of motif 1 and motif 2 was observed in all *Aspergillus* GATA TFs; however, the compositions of GATA TF motifs also contained different variable regions. As expected, GATA members that had similar motif compositions could be clustered into one subgroup, which suggested they may perform similar genetic functions within the same subgroups. In addition, the motif distribution further confirmed the accuracy of the phylogenetic relationship of *Aspergillus* GATA TFs. The distribution of motifs in different subgroups indicated functional differentiation in GATA TFs during evolution.

### Effects of different temperature and salinity treatments on the growth of *A. oryzae*

**Fig. 4:**
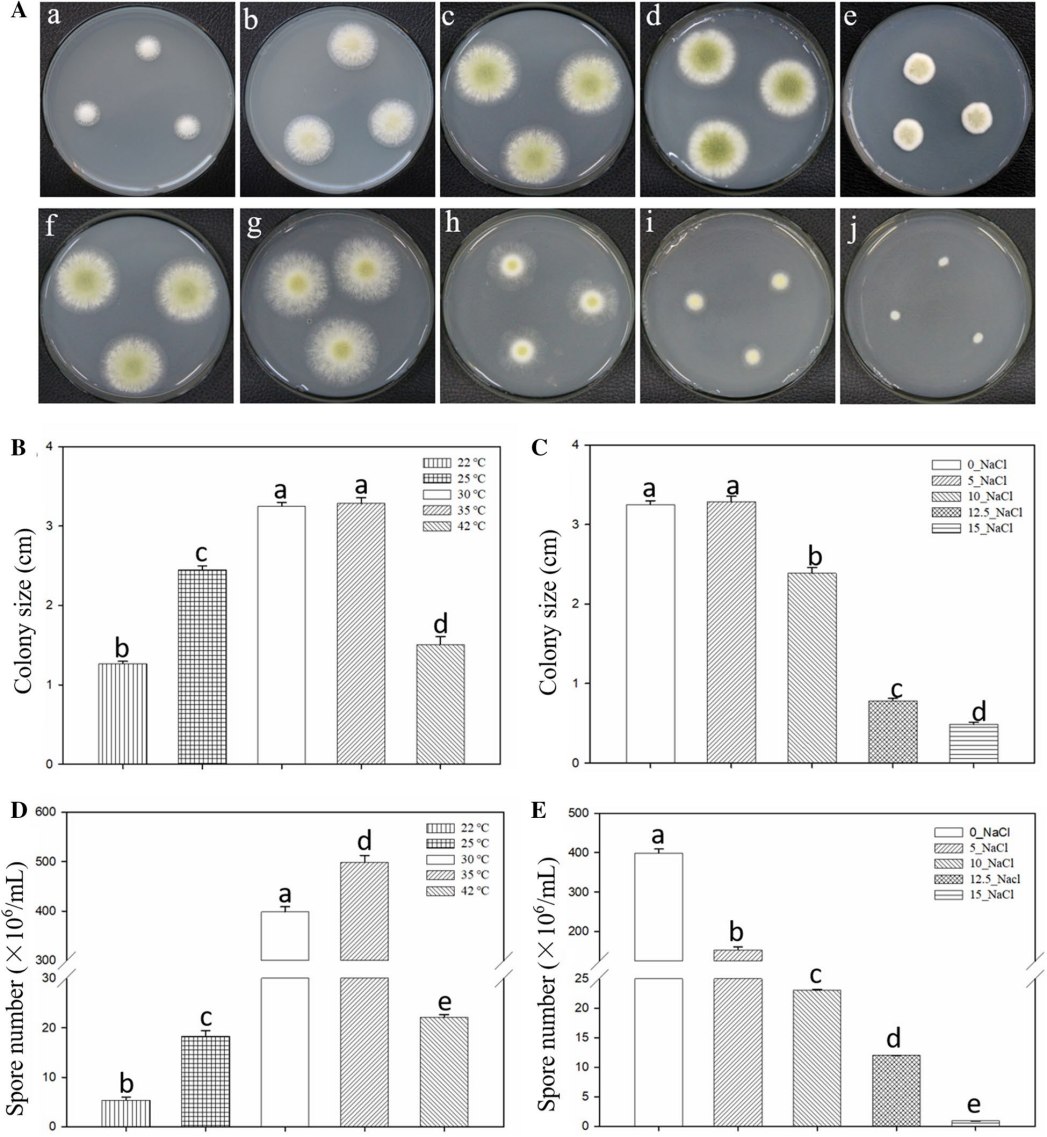
*A. oryzae* hyphal growth and differentiation under different stress factors for 72 h. **a** The phenotypes of *A.* *oryzae* under temperature and salinity stress. (a–e) Phenotypes of *A.* *oryzae* exposed to different temperature stresses (22, 25, 30, 35, and 42 ℃ from left to right). (f-j) 0.0, 5.0, 10.0, 12.5, and 15.0 g/100 mL NaCl was used for salinity stress. **b** and **c** Colony diameter was determined by measuring diameter under different stress conditions. **d** and **e** Spore numbers were counted under temperature and salinity stresses. The spores were collected from colonies and suspended in sterile distilled water to obtain the spore suspension, and then the spore numbers were counted using a hemocytometer. The optimum growth temperature of *A.* *oryzae* (30 ℃), was used as the control temperature in the experiment. The PDA medium without NaCl used as the control in the salt treatment experiments. Results represent the average of three repetitions ± SEM (n = 3). Different letters in the bar chart represent significant differences (p < 0.01, Duncan's multiple range test); the same letters in the bar chart represent absence of significant difference when compared to the control

Temperature and salt concentration are two of the most important environmental factors affecting the growth and fermentation of *A.* *oryzae* during fermentation (Chen et al. 2011; Bechman et al. 2012; Wang et al. 2013). Therefore, we investigated the growth of *A. oryzae* under different temperature and salt concentration stresses. The optimum temperature for *A.* *oryzae* growth usually ranges from 30 to 35 °C. Low and high temperatures significantly inhibited mycelial growth, especially at the temperature of 22 and 42 ℃ (Fig. 4a(a–e), b). In addition, high salt concentration also significantly inhibited hyphal growth and differentiation of *A.* *oryzae*, and the inhibitory effect increased with salt concentration (Fig. 4a(f–j), c). Furthermore, the formation and development of *A. oryzae* spores, which shows yellow-green color in the middle of the fungal colony, were also inhibited under low- and high-temperature and high salinity stresses (Fig. 4a, d and e).

### Expression patterns of *A. oryzae* GATA TFs in response to temperature and salinity stresses

**Fig. 5:**
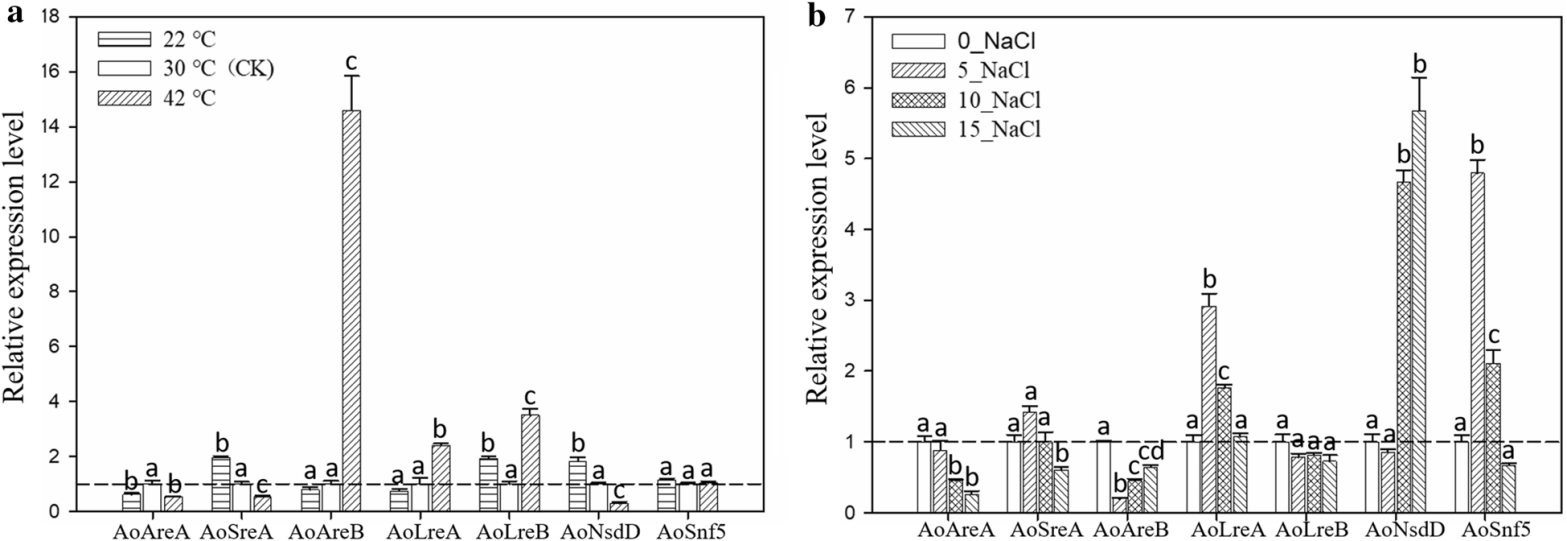
Expression levels of *A. oryzae* GATA TFs in response to temperature and salt stresses. **a** The relative expression levels of *A.* *oryzae* GATA TFs responding to low- and high-temperature stresses. **b** The expression patterns of *A.* *oryzae* GATA TFs under different salt concentration stresses. The optimum growth temperature of *A.* *oryzae* (30 ℃) was used as the control temperature (CK) in the experiment. PDA medium without NaCl was used as the control (CK) under salt stress. Results show the average of three repetitions ± SEM (n = 3). Different letters represent significant differences (p < 0.01, Duncan's multiple range test); same letters represent lack of significant difference when compared to the control

To determine the roles of *A. oryzae* GATA TFs in response to abiotic stresses, we analyzed the expression level of the seven *A.* *oryzae* GATA TFs using qRT-PCR in *A.* *oryzae* that grew at different temperatures and salt concentrations (Fig. 5). and observed that their expression varied under different temperatures and salt stresses. With the exception of the *AoSnf5*, the six other *A.* *oryzae* GATA TFs strongly responded to low or high temperatures (Fig. 5a). *AoSreA* and *AoNsdD* showed the same expression trend, as they were significantly induced at low temperature (22 ℃) and inhibited at high temperature (42 ℃) compared with CK (30 ℃). In addition, *AoAreB*, *AoLreA*, and *AoLreB*, especially *AoAreB*, were remarkably upregulated at high temperature compared to CK (30 ℃) (Fig. 5a). Interestingly, only *AoAreA* was inhibited at both low and high temperature. Furthermore, *AoAreA*, *AoSreA*, and *AoAreB* were significantly downregulated under high-salt concentration stress, while *AoLreA*, *AoNsdD*, and *AoSnf5* were upregulated in the presence of 5.0 and 10.0 g/100 mL NaCl (Fig. 5b). In addition, we compared the results of qRT-PCR analysis with the transcriptional data of the seven TFs in response to salt stress. We observed strong correlation between the results of qRT-PCR and those obtained using RNA-seq (Pearson correlation, *R*^2^ = 0.9044 (Additional file 5: Figure S2). Together, the results highlighted the importance of *A.* *oryzae* GATA TFs in response to temperature and high salt stresses and provided a basis for future studies into the function of *A.* *oryzae* GATA in abiotic stresses.

### PPI network of *A. oryzae* GATA TFs

**Fig. 6:**
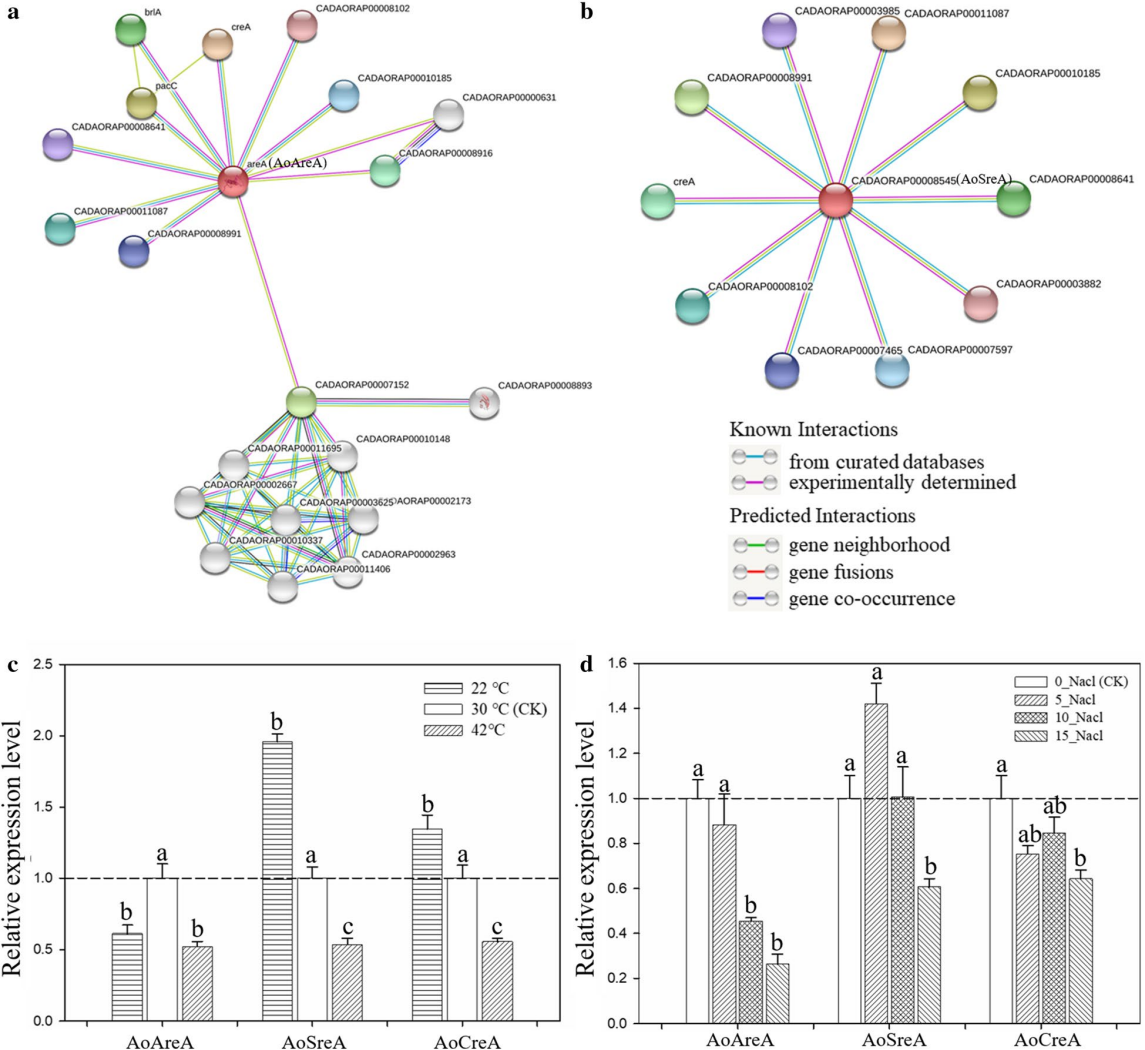
Protein–protein interaction (PPI) network of *A. oryzae* GATA TFs. **a**, **b** The PPI network of AoAreA and AoSreA. (C, D) The relative expression levels of *AoAreA* and *AoSreA* were consistent with that of the interaction partner, *AoCreA* (p < 0.01, n = 3). The optimum growth temperature of *A*. *oryzae* (30 ℃) was used as the control temperature (CK) in the experiment. PDA medium without NaCl was used as the control (CK) under salt stress. The same letters represent lack of significant difference compared to the control when assessed using Duncan's multiple range test

To analyze the functions of *A.* *oryzae* GATA TFs, a PPI network was constructed using the data from the STRING database, and only two independent PPI network of AoAreA and AoSreA was obtained (Fig. 6a, b). Furthermore, we observed that both AoAreA and AoSreA interacted with CreA, and that *CreA* deletion mutants showed less conidiation than the wild type and sensitivity to salt stress (Hou et al. 2018). Therefore, the expression levels of *AoAreA*, *AoSreA*, and *AoCreA* were analyzed under temperature and salt stresses. *AoSreA* and *AoCreA* showed the same expression patterns under both low and high temperature stresses, while *AoAreA* and *AoCreA* exhibited opposite expression level at 22 ℃ (Fig. 6c). Interestingly, these three genes showed the same expression patterns under high salt concentration stress (Fig. 6d), which demonstrates that *AoCreA* may be positively coregulated by both *AoAreA* and *AoSreA* under salt stresses. Additionally, glutathione S-transferase (CADAORAP00007152), which is critical to abiotic stress was also found in the network of *AoAreA* (Favaloro et al. 2000). These results will be beneficial for identifying important proteins and biological modules that interact with *A. oryzae* GATA TFs and understanding the roles of *A.* *oryzae* GATA TFs in response to abiotic stresses. The detailed information regarding the proteins in the PPI network is listed in Additional file 3: Table S3.

## Discussion

Transcription factors (TFs) regulate expression of genes that mediate growth processes and environmental response and are employed as a principal source of the diversity and change that underlie evolution (Riechmann and Ratcliffe 2000). Fungal GATA TFs are mainly involved in nitrogen metabolism (Michielse et al. 2014; Pfannmüller et al. 2017), light responses (Purschwitz et al. 2008; Fuller et al. 2013), siderophore biosynthesis and mating-type switching (Jung and Kronstad 2011). Few fungal GATA TFs, such as the *SreA*, *SreB*, *LreA*, *LreB*, *GLN3*, and *GAT1*, also participate in response to abiotic stresses, (Chung et al. 2020; Crespo et al. 2001; Purschwitz et al. 2008; Fuller et al. 2013; Marty et al. 2015). In this study, we focused on the GATA TF family in *A*. *oryzae* *3.042* to define the genetic characteristics and improve our understanding regarding their role in response to temperature and salinity stresses. Seven GATA TFs were identified from the *A.* *oryzae 3.042* genome using an HMM model, which was similar to the number of GATA TFs identified in the model fungi, *Fusarium graminearum* and *Botrytis* *cinerea*, which contain 7 and 7 GATA TFs, respectively (Zhang et al. 2014). The number of the GATA TFs is conserved among *A*. *clavatus*, *A*. fla*vus*, *A*. *fumigatus*, *A*. *nidulans*, and *A*. *niger* that possess six GATA TFs, suggesting that the composition of GATA TFs in filamentous fungi is identical (Kim et al. 2006; Kobayashi et al. 2007). Interestingly, *A*. *oryzae* contains one more GATA TF (*AoSnf5*) compared to these *Aspergillus*. Furthermore, we also detected the homologous gene of *AoSnf5* in the genomes of *Aspergillus* species, which was annotated as unnamed or hypothetical protein; however, *AoSnf5*, encoding a GATA protein with 20 residues between the Cys-X_2_-Cys motifs, was identified as an *Aspergillus* GATA TF for the first time.

Although most GATA domains harbor a class-IV zinc-finger motif, their structure varies among kingdoms (Lowry and Atchley 2000). In plants, most GATA domains have a single Cys-X_2_-Cys-X_18_-Cys-X_2_-Cys motif, but some harbor more than two zinc-finger motifs or 20 residues within zinc-finger loops (Reyes et al. 2004; Behringer and Schwechheimer 2015). In animals, the GATA domain harbors two zinc-finger motifs with Cys-X_2_-Cys-X_17_-Cys-X_2_-Cys, but only the C-terminal finger is associated with DNA binding (Patient and Mcghee 2002). Fungal GATA TFs are combination of both animal and plant GATA TFs in terms of the amino acid residues present in the zinc-finger loop (Teakle and Gilmartin 1998). The majority of fungal GATA TFs contain a single zinc-finger domain and fall into two different categories: animal-like with 17-residue loops (Cys-X_2_-Cys-X_17_-Cys-X_2_-Cys), and plant-like with 18-residue loops (Cys-X_2_-Cys-X_18_-Cys-X_2_-Cys) (Teakle and Gilmartin 1998; Scazzocchio 2000; Patient and Mcghee 2002). Nineteen- and 20-residue zinc-finger loops (Cys-X_2_-Cys-X_19-20_-Cys-X_2_-Cys) are also found in fungi, albeit rarely (Scazzocchio 2000; Maxon and Herskowitz 2001). With the exception of the 17- and 18-residue zinc-finger loops in *A.* *oryzae* GATA TFs, the novel *AoSnf5* contains 20 residues in the zinc-finger loops (Cys-X_2_-Cys-X_20_-Cys-X_2_-Cys), which are rarely found in fungi (Table1 and Fig. 1). To the best of our knowledge, this is the first study to identify GATA TFs with 20-residue zinc-finger loops in *Aspergillus*. In addition, *AoSreA* harbors two ZnF-GATA domains of Cys-X_2_-Cys-X_17_-Cys-X_2_-Cys type, which is the typical GATA characteristic of animals (Lowry and Atchley 2000; Patient and Mcghee 2002). Therefore, the features of *A.* *oryzae* GATA TFs demonstrated that *A.* *oryzae* GATA TFs might be the combination of both plant and animal GATA TFs, which is consistent with the report showing that fungal GATA TFs are combinations of both plant and animal GATA TFs in terms of the numbers of ZnF-GATA domains and amino acid residues present in the zinc-finger loop.

In the phylogenetic tree, seven *A*. *oryzae* GATA proteins and other *Aspergillus* GATA TFs were classified into seven subgroups. Consistent with the report of Kobayashi et al. (2007), six known *A.* *oryzae* GATA TFs were classified into six functional subgroups based on the number of ZnF_GATA domains and zinc finger motifs of GATA domain sequences with other *Aspergillus* GATA TFs from FTFD in the phylogenetic NJ tree. In addition, 11 *Aspergillus* GATA TFs clustered together in an unnamed subgroup (Fig. 2). The phylogenetic positions of fungal GATA TFs are different from those of animal and plant GATA TFs, and most fall into independent clusters. Fungal GATA TFs can be divided into seven phylogenetic subgroups named SRP, NIT2, ASD4, WC1, WC2, NSSDD, and SFH1(Kim et al. 2006; Yu et al. 2019). In this study, all the *Aspergillus* GATA TFs were divided into seven subgroups consistent with the phylogenetic analysis of fungal GATA TFs, and the unnamed subgroup of *Aspergillus* GATA TFs might correspond to the SNF1 subgroup in the NJ tree. The GATA TFs of the SFH1 subgroup contain a SNF5/SMARCB1/INI1 domain characteristic of the Swi/Snf family of chromatin remodeling complex in *S*. *cerevisiae* (Klochendler-Yeivin and Yaniv 2001). The biological functions of SFH1 GATA factors in filamentous ascomycetes have not yet been characterized. Interestingly, AoSnf5, a newly identified GATA TF in our study, also contained a SNF5 domain similar to that present in the SFH1 subgroup GATA TFs; however, AoSnf5 clustered in NSDD subgroup together with AoNsdD (Fig. 2). Although we cannot explain this phenomenon yet, the identification of AoSnf5 GATA TF enriches the *Aspergillus* GATA TF family. Additionally, conserved motifs demonstrated that GATA TF members with similar motif compositions could be clustered into one subgroup (Fig. 3), indicating that they may perform similar genetic functions within the same subgroups. In addition, the motif distribution further confirms the accuracy of the phylogenetic relationship of *Aspergillus* GATA TFs. The analyses of the phylogenetic tree and conserved motifs demonstrated that the GATA TFs among different *Aspergillus* were evolutionarily conserved and performed similar function within the same subgroups.

Although the GATA motif and DNA binding specificity were conserved, the rest of the protein was not, thereby leading to the same motif serving different purposes in various contexts. Deletion of *A*. *nidulans AreB* has significant phenotypic effects on the utilization of specific carbon sources, confirming its role in the regulation of carbon metabolism. *AreB* is shown to regulate the expression of *AreA* regulatory gene suggesting *AreB* has a range of indirect regulatory effects (Chudzicka-Ormaniec et al. 2019). The functional difference between *AreB* and *AreA* mainly because of the difference in the component of conserved motifs (Fig. 3). The deletion of GATA *gzf1* did not display a growth defect on any of the nitrogen sources tested in *Yarrowia lipolytica*, which is homologous to nitrogen starvation response gene *A*. *nidulans AreA* (Pomraning et al. 2017). Therefore, specific motif and structures contained in *A*. *oryzae* might perform functions related to special biological processes.

TFs are the key transcriptional regulators that exhibit different expression profiles under distinct physiological and environmental conditions and synchronize stimuli and response. Many studies have revealed the GATA TFs are involved in the regulation of various abiotic stress responses in plants (Peng et al. 2015; Gupta et al. 2017; Nutan et al. 2019) and few fungi (Crespo et al. 2001; Fuller et al. 2013; Marty et al. 2015; Chung et al. 2020). Temperature and salt concentration are two of the most important environmental factors affecting the growth of *A.* *oryzae* during fermentation (Machida et al. 2018; Chen et al. 2011; Bechman et al. 2012; Wang et al. 2013). *AreA* and *AreB* function as positive and negative transcriptional regulators that regulat nitrogen and carbon metabolism in *Fusarium fujikuroi* and *A.* *nidulans* (Michielse et al.^2014^; Pfannmüller et al. 2017; Chudzicka-Ormaniec et al. 2019). The expression level of *AoAreA* and *AoAreB* showed opposite trends at high temperature (42 ℃) compared to that of CK (30 ℃) in *A.* *oryzae* (Fig. 5a), which indicated *AoAreA* and *AoAreB* might also act as negative and positive transcriptional regulators under high-temperature stress, respectively. The clustering of AoNsdD and AoSnf5 in the NSDD subgroup in the NJ tree (Fig. 2) was strongly induced under high salt stress. *NsdD* has been reported to be a key repressor affecting the quantity of asexual spores in *Aspergillus* (Lee et al. 2014, 2016), although studies regarding the response of *NsdD* to adversity stress in *Aspergillus* are lacking. In addition to the regulation of siderophore biosynthesis and iron metabolism, *SreA* is also related to the maintenance of cell wall integrity and negatively impacts resistance, as Δ*sreA* increases resistance to H_2_O_2_, calcofluor white, and Congo red (Chung et al. 2020). *AoSreA* was significantly downregulated at 42 ℃ and under high salt stress, which indicates that *AoSreA* might negatively impact high temperature and high salt resistance. In contrast, *AoSreA* was significantly upregulated at 22 ℃, and a report shows that the *SreB* strongly expresses and contributes to filamentous growth at 22 ℃ via lipid metabolism in *Blastomyces dermatitidis* (Marty et al. 2015). The ZnF_GATA domain is conserved in *AoSreA* and *SreB* (Additional file 6: Figure S3), which demonstrates that overexpression *AoSreA* in *A. oryzae* might also enhance the growth of mycelium at 22 ℃. Furthermore, *AoCreA*, protein of which interacts with AoSreA in the PPI network, has the same expression pattern as *AoSreA*, indicating that *AoSreA* might positively regulate *AoCreA* under temperature and high salt stresses. Interestingly, *AoCreA* expression was suppressed under high salt stress in *A. oryzae*, which is in contrast to the results of a previous study showing that Δ*creA* mutants of *Fusarium graminearum* are sensitive to salt stress (Hou and Wang 2018). However, the results provide insights regarding the critical role of *SreA* in resistance to different temperatures and high salt stresses in *A.* *oryzae*.

*LreA* and *LreB*, the GATA TFs of WC1 and WC2 subgroups, are involved in the regulation of blue- and red-light responses (Purschwitz et al. 2008; Fuller et al.^2013^). *AoLreA* and *AoLreB*, belonging to WC1 and WC2 subgroups in the NJ tree (Fig. 2), act as a dimer and contain typical PAS dimerization domains shown in Table 1 and Fig. 1b. Previous studies have demonstrated that the PAS domain also functions in sensing environmental or physiological signals including oxidative and heat stress (Nan et al. 2011; Corrada et al. 2016). Therefore, except for the regulation of blue- and red-light responses, the PAS domains present in *AoLreA* and *AoLreB* may facilitate the environmental response of *A.* *oryzae* GATA TFs. Additionally, *LreA* and *LreB* is a regulatory complex of the global regulator *VeA*, which plays a critical role in environmental stress response in *A.* *cristatus*; indeed, the Δ*veA* mutants are more sensitive to high salt, osmotic pressure, and temperature stress (Calvo 2008; Tan et al. 2018). In our study, *AoLreA* and *AoLreB* expression increased under high-temperature (42 ℃) stress, and *AoLreA* expression was significantly induced in the presence of 5.0 and 10.0 g/100 mL NaCl. These results demonstrated that *AoLreA* and *AoLreB* might act as a regulatory complex of the global regulator *VeA* in response to temperature and high salt stresses in *A.oryzae*.

In summary, we identified and functionally characterized seven GATA TFs proteins from *A*. *oryzae* 3.042 genome. Compared to the previous reports, *A*. *oryzae* contains one more GATA TF (AoSnf5), which encodes a GATA protein with 20 residues between the Cys-X_2_-Cys motifs. To the best of our knowledge, this is the first study to identify GATA TFs with 20-residue zinc-finger loops in *Aspergillus*. Our results may be useful for elucidating the evolutionary relationships, expression patterns, and functional divergence of GATA TFs in *A.* *oryzae* and enrich the *Aspergillus* GATA TF family. In addition, the expression patterns of these *A.* *oryzae* GATA TFs under distinct environmental conditions provided useful information for further analysis of the function of *A.* *oryzae* GATA TFs in regulation of various abiotic stress responses in *Aspergillus.*

## Supplementary Information

**Additional file 1**:** Table S1**. Sequence IDs of GATA TFs used to construct the neighbor-joining phylogenetic tree.

**Additional file 2**:** Table S2**. Primers used in qRT-PCR of *A*. *oryzae* GATA gene in response to abiotic stress.

**Additional file 3**:** Table S3**. Detailed information regarding the proteins in the PPI network.

**Additional file 4**:** Figure S1**. The five structural motifs in *A*. *oryzae* GATA TF proteins.

**Additional file 5**:** Figure S2**. The expression correlation of GATA TFs between the qRT-PCR results and those obtained using RNA-seq.

**Additional file 6**:** Figure S3**. Alignment of the predicted amino acid sequence of AoSreA with SreB. AoSreA and SreB contained several conserved domains including two ZnF_GATA (N-terminal and C-terminal) separated by a cysteine-rich region (CRR) and a conserved C-terminus (CCT) with a predicted coiled-coil domain.

## Data Availability

The genome-wide transcriptome data of *A. oryzae* in different growth stages and salt stress treatment have been submitted to NCBI SRA databases under Bioproject Accession PRJNA407002 and PRJNA383095.

## Abbreviations

TF: Transcription factors
NJ tree: Neighbor-joining phylogenetic tree
PPI: Protein–protein interaction network
PDA: Potato dextrose agar
