# Supplementary material for: Genome-wide identification of the GATA transcription factor family and their expression patterns under temperature and salt stress in Aspergillus oryzae
Source: AMB Express. 2021 Apr 19;11:56. doi: 10.1186/s13568-021-01212-w (PMC8055810; doi:10.1186/s13568-021-01212-w)
Supplement: Supplementary file 1 — Additional file 1: Table S1. Sequence IDs of GATA TFs used to construct the neighbor-joining phylogenetic tree. [file 13568_2021_1212_MOESM1_ESM.docx]

| **Table S1 Sequence IDs of GATA TFs used to construct the** **neighbor-joining phylogenetic tree.** | | |
| --- | --- | --- |
| Species | GATA ID in FTFD | GATA ID in NCBI |
| *Aspergillus clavatus NRRL 1* | Acl0006 (ACLA_072190) | XP_001275612.1 |
| *Aspergillus clavatus NRRL 1* | Acl0002 (ACLA_015750) | XP_001274559.1 |
| *Aspergillus clavatus NRRL 1* | Acl0007 (ACLA_098570) | XP_001270341.1 |
| *Aspergillus clavatus NRRL 1* | Acl0004 (ACLA_041500) | XP_001271358.1 |
| *Aspergillus clavatus NRRL 1* | Acl0001 (ACLA_014320) | XP_001274421.1 |
| *Aspergillus clavatus NRRL 1* | Acl0003 (ACLA_033760) | XP_001270599.1 |
| *Aspergillus clavatus NRRL 1* | Acl0013 (ACLA_072190) | XP_001275612.1 |
| *Aspergillus clavatus NRRL 1* | Acl0008 (ACLA_014320) | XP_001274421.1 |
| *Aspergillus clavatus NRRL 1* | Acl0011 (ACLA_041500) | XP_001271358.1 |
| *Aspergillus clavatus NRRL 1* | Acl0010 (ACLA_033760) | XP_001270599.1 |
| *Aspergillus clavatus NRRL 1* | Acl0009 (ACLA_015750) | XP_001274559.1 |
| *Aspergillus clavatus NRRL 1* | Acl0012 (ACLA_051670) | XP_001272121.1 |
| *Aspergillus clavatus NRRL 1* | Acl0014 (ACLA_098570) | XP_001270341.1 |
| *Aspergillus flavus* | Afl0006 (AFL_07080) | KAF7630234.1 |
| *Aspergillus flavus* | Afl0007 (AFL_08367) | XP_002376041.1 |
| *Aspergillus flavus* | Afl0005 (AFL_06436) | KOC08900.1 |
| *Aspergillus flavus* | Afl0003 (AFL_04644) | XP_002379623.1 |
| *Aspergillus flavus* | Afl0001 (AFL_04028) | KAB8245227.1 |
| *Aspergillus flavus* | Afl0004 (AFL_05957) | QMW25010.1 |
| *Aspergillus flavus* | Afl0011 (AFL2G_08402) | QMW33228.1 |
| *Aspergillus flavus* | Afl0010 (AFL2G_06945) | XP_002379623.1 |
| *Aspergillus flavus* | Afl0008 (AFL2G_03635) | XP_002376041.1 |
| *Aspergillus flavus* | Afl0012 (AFL2G_10206) | RAQ50831.1 |
| *Aspergillus flavus* | Afl0013 (AFL2G_10376) | QMW25010.1 |
| *Aspergillus flavus* | Afl0014 (AFL2G_11225) | KAB8245227.1 |
| *Aspergillus flavus* | Afl0009 (AFL2G_06596) | XP_002379258.1 |
| *Aspergillus fumigatus A1193* | Af10005 (EDP52370.1) | EDP53155.1 |
| *Aspergillus fumigatus A1193* | Af10006 (EDP53155.1) | EDP53155.1 |
| *Aspergillus fumigatus A1193* | Af10007 (EDP54843.1) | XP_755671.1 |
| *Aspergillus fumigatus A1193* | Af10001 (EDP47785.1) | EDP47785.1 |
| *Aspergillus fumigatus A1193* | Af10003 (EDP51862.1) | XP_753523.1 |
| *Aspergillus fumigatus A1193* | Af10004 (EDP52028.1) | XP_753363.1 |
| *Aspergillus fumigatus A1193* | Af10002 (EDP50624.1) | EDP50624.1 |
| *Aspergillus fumigatus* | Af0006 (Afu5g12900) | XP_753363.1 |
| *Aspergillus fumigatus* | Af0012 (Afu5g11260) | XP_753523.1 |
| *Aspergillus fumigatus* | Af0011 (Afu4g12690) | XP_751563.1 |
| *Aspergillus fumigatus* | Af0010 (Afu3g13870) | XPRAQ50831.1_754237.1 |
| *Aspergillus fumigatus* | Af0014 (Afu6g01970) | XP_747880.1 |
| *Aspergillus fumigatus* | Af0013 (Afu5g12900) | XP_753363.1 |
| *Aspergillus fumigatus* | Af0009 (Afu3g05780) | XP_755026.1 |
| *Aspergillus nidulans FGSC A4* | An0004 (ANID_03607) | XP_661211.1 |
| *Aspergillus nidulans FGSC A4* | An0007 (ANID_08667) | XP_681936.1 |
| *Aspergillus nidulans FGSC A4* | An0006 (ANID_06828) | XP_664432.1 |
| *Aspergillus nidulans FGSC A4* | An0005 (ANID_06221) | XP_663825.1 |
| *Aspergillus nidulans FGSC A4* | An0002 (ANID_03152) | XP_660756.1 |
| *Aspergillus nidulans FGSC A4* | An0003 (ANID_03436) | XP_661040.1 |
| *Aspergillus nidulans FGSC A4* | An0001 (ANID_00176) | XP_657780.1 |
| *Aspergillus nidulans* | An0007 (AN8667.3) | XP_681936.1 |
| *Aspergillus nidulans* | An0005 (AN6221.3) | AAG49351.1 |
| *Aspergillus nidulans* | An0004 (AN3607.3) | XP_661211.1 |
| *Aspergillus nidulans* | An0006 (AN6828.3) | XP_026599249.1 |
| *Aspergillus niger ATCC 1015* | AnA0001 (estExt_fgenesh1_pg.C_10946) | XP_001388675.1 |
| *Aspergillus niger ATCC 1015* | AnA0005 (fgenesh1_pg.C_scaffold_2000168) | XP_001399365.2 |
| *Aspergillus niger ATCC 1015* | AnA0003 (estExt_fgenesh1_pg.C_90054) | XP_025450915.1 |
| *Aspergillus niger ATCC 1015* | AnA0006 (fgenesh1_pg.C_scaffold_2000697) | XP_025459789.1 |
| *Aspergillus niger ATCC 1015* | AnA0007 (gw1.4.98.1) | EHA20102.1 |
| *Aspergillus niger ATCC 1015* | AnA0002 (estExt_fgenesh1_pg.C_80499) | EHA28021.1 |
| *Aspergillus niger CBS 513.88* | [AnC0004 (An02g09610)](http://ftfd.snu.ac.kr/tf.php?a=tf_sequence&id=189869&p=&l=&sf=&sv=) | CAK37830.2 |
| *Aspergillus niger CBS 513.88* | [AnC0003 (An02g02240)](http://ftfd.snu.ac.kr/tf.php?a=tf_sequence&id=189870&p=&l=&sf=&sv=) | CAK96398.1 |
| *Aspergillus niger CBS 513.88* | [AnC0002 (An01g08210)](http://ftfd.snu.ac.kr/tf.php?a=tf_sequence&id=189871&p=&l=&sf=&sv=) | CAK43913.1 |
| *Aspergillus niger CBS 513.88* | [AnC0001 (An01g02370)](http://ftfd.snu.ac.kr/tf.php?a=tf_sequence&id=189872&p=&l=&sf=&sv=) | XP_001388675.1 |
| *Aspergillus niger CBS 513.88* | [AnC0006 (An12g08960)](http://ftfd.snu.ac.kr/tf.php?a=tf_sequence&id=189873&p=&l=&sf=&sv=) | CAK46383.1 |
| *Aspergillus niger CBS 513.88* | [AnC0007 (An14g06470)](http://ftfd.snu.ac.kr/tf.php?a=tf_sequence&id=189874&p=&l=&sf=&sv=) | XP_001401270.1 |
| *Aspergillus niger CBS 513.88* | [AnC0005 (An11g10820)](http://ftfd.snu.ac.kr/tf.php?a=tf_sequence&id=189875&p=&l=&sf=&sv=) | XP_001395039.1 |
| *Aspergillus terreus* | Ate0005 (ATEG_07714.1) | XP_001216335.1 |
| *Aspergillus terreus* | Ate0004 (ATEG_07264.1) | XP_001209950.1 |
| *Aspergillus terreus* | Ate0003 (ATEG_04043.1) | XP_001213221.1 |
| *Aspergillus terreus* | Ate0002 (ATEG_03152.1) | XP_001212330.1 |
| *Aspergillus terreus* | Ate0006 (ATEG_08959.1) | XP_001217545.1 |
| *Aspergillus terreus* | Ate0001 (ATEG_01997.1) | XP_001211175.1 |
| *Aspergillus oryzae  3.042* |  | EIT82081.1AoSreA |
| *Aspergillus oryzae  3.042* |  | EIT79032.1AoAreB |
| *Aspergillus oryzae  3.042* |  | EIT72728.1AoAreA |
| *Aspergillus oryzae  3.042* |  | EIT79273.1AoLreB |
| *Aspergillus oryzae  3.042* |  | EIT79449.1AoNsdD |
| *Aspergillus oryzae  3.042* |  | EIT77832.1AoLreA |
| *Aspergillus oryzae  3.042* |  | EIT78280.1AoSnf5 |
