# Supplementary material for: Genome-wide identification of the GATA transcription factor family and their expression patterns under temperature and salt stress in Aspergillus oryzae
Source: AMB Express. 2021 Apr 19;11:56. doi: 10.1186/s13568-021-01212-w (PMC8055810; doi:10.1186/s13568-021-01212-w)
Supplement: Supplementary file 2 — Additional file 2: Table S2. Primers used in qRT-PCR of A. oryzae GATA gene in response to abiotic stress. [file 13568_2021_1212_MOESM2_ESM.docx]

| **Table S2 qRT-PCR primers of *A. oryzae* GATA gene expression in response to abiotic stress.** | | |
| --- | --- | --- |
| Genes | Primer sequence 5'→3' | product size (bp) |
| AoAreA | F: ACTTTTACCACCCACCACCC; R: CTGAAGTAGGCCGTCATGGG | 144 |
| AoSnf5 | F: TCCGCGAGTCCGTCAATATG; R: TATTGCTGCTGTTCCTCGCT | 103 |
| AoLreB | F: GCTCAGGAAATGGTGGCTCT; R: ACTTCTCGTCCAATGCTGGG | 113 |
| AoLreA | F: GCAGGGAGGCAATGATGAGT; R: TCCCGCTACACCAGAAACAC | 106 |
| AoSreA | F: TCGGTAAAGAATGGCACCCC; R: ATTCTCGTCTCGTCGCCAAA | 108 |
| AoNsdD | F: CATCCACGAGGCGTTAGGAA; R: ACAGGAGATGTGAATGCGGT | 101 |
| AoAreB | F: CTCCCGTGTGCCAAAATTGC; R: GCTTATCGGACGGGGTCTTC | 125 |
| AoCreA | F: CGGTCACATGCGTTCCAATC; R: TGGTGATTACGTGGACCAGC | 150 |
| RH-actin | F: GACAACATCCAGGGTATCACTAAGC;  R: GGTCTCCTCGTAGATCATGGCA | 140 |
| F: Forward primer, R: Reverse primer; RH-actin: Reference gene in qRT-PCR analysis | | |
