# Supplementary material for: Genome-wide identification of the GATA transcription factor family and their expression patterns under temperature and salt stress in Aspergillus oryzae
Source: AMB Express. 2021 Apr 19;11:56. doi: 10.1186/s13568-021-01212-w (PMC8055810; doi:10.1186/s13568-021-01212-w)
Supplement: Supplementary file 3 — Additional file 3: Table S3. Detailed information regarding the proteins in the PPI network. [file 13568_2021_1212_MOESM3_ESM.docx]

| Table S3 The detailed information of the proteins in the PPI network. | |
| --- | --- |
| Predicted Functional Partners | Information of Predicted Functional Partners |
| The protein in PPI network of *AoAreA* | |
| creA | DNA-binding protein creA |
| pacC | PH-response transcription factor pacC/RIM101 |
| CADAORAP00007152 | Glutathione S-transferase |
| blrA | BRLA predicted protein |
| CADAORAP00008916 | Ammonia protein |
| CADAORAP00011087 | Predicted protein |
| CADAORAP00010185 | Zn-finger |
| CADAORAP00008991 | Zn-finger |
| CADAORAP00008641 | Predicted protein |
| CADAORAP00008102 | Predicted protein |
| The protein in PPI network of *AoSreA* | |
| CADAORAP00011087 | Predicted protein |
| CADAORAP00010185 | Zn-finger |
| CADAORAP00008991 | Zn-finger |
| CADAORAP00008641 | Predicted protein |
| creA | DNA-binding protein creA |
| CADAORAP00008102 | Predicted protein |
| CADAORAP00007597 | Predicted protein |
| CADAORAP00007465 | Predicted protein |
| CADAORAP00003985 | Predicted protein |
| CADAORAP00003882 | Predicted protein |
