# Supplementary figures and images for: Genome-wide identification of the GATA transcription factor family and their expression patterns under temperature and salt stress in Aspergillus oryzae
Source: AMB Express. 2021 Apr 19;11:56. doi: 10.1186/s13568-021-01212-w (PMC8055810; doi:10.1186/s13568-021-01212-w)

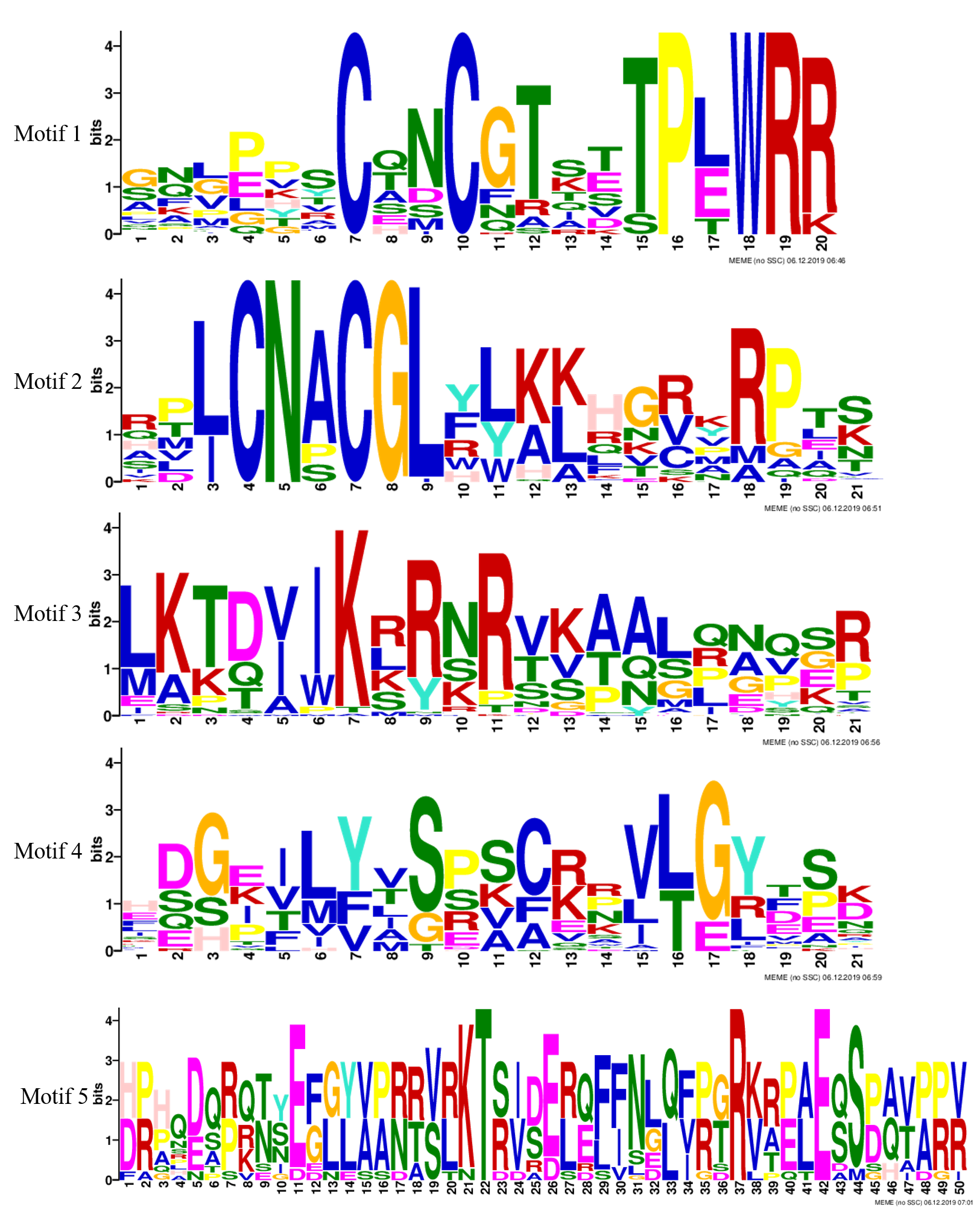

Supplement: Supplementary file 4 — Additional file 4: Figure S1. The five structural motifs in A. oryzae GATA TF proteins. [file 13568_2021_1212_MOESM4_ESM.tif]

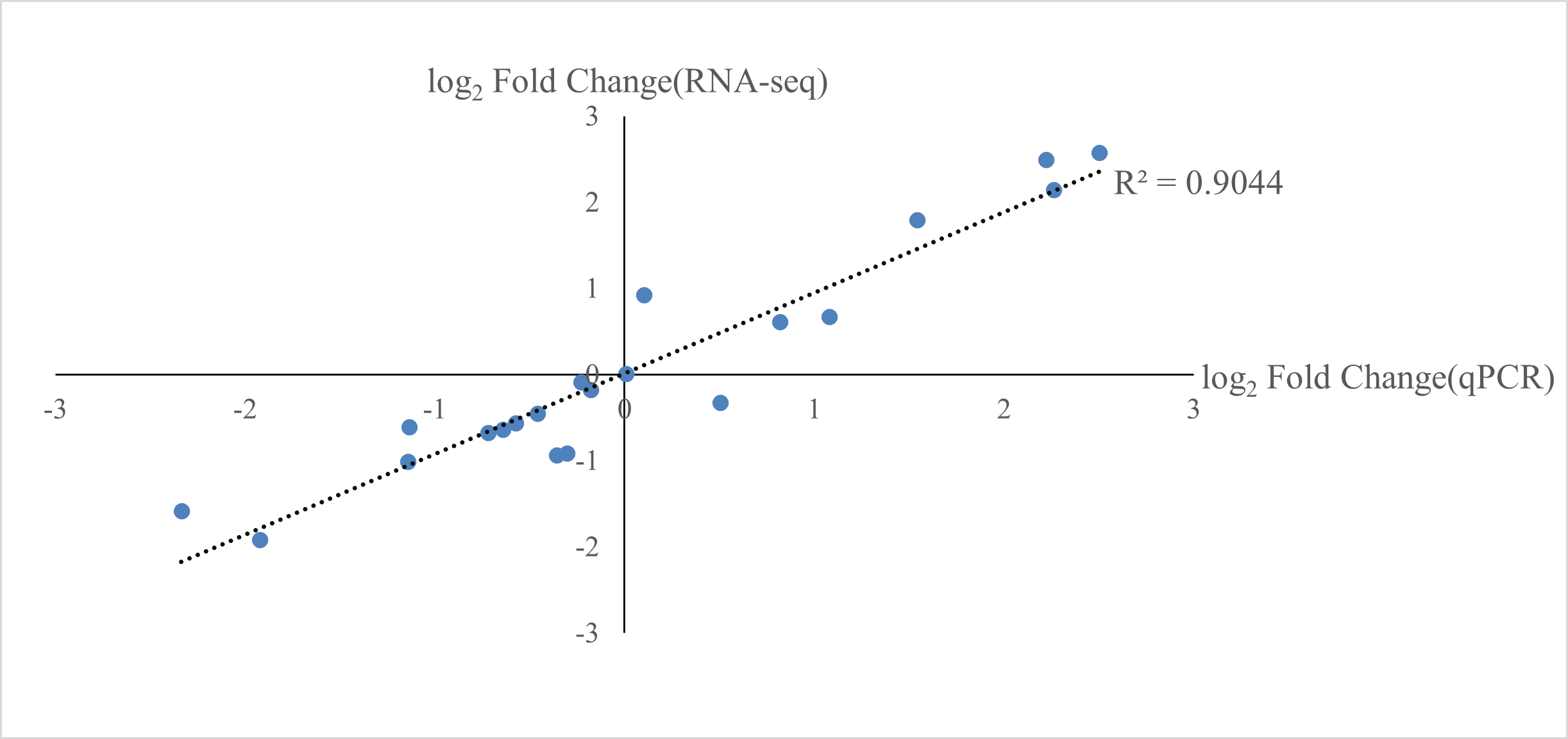

Supplement: Supplementary file 5 — Additional file 5: Figure S2. The expression correlation of GATA TFs between the qRT-PCR results and those obtained using RNA-seq. [file 13568_2021_1212_MOESM5_ESM.tif]

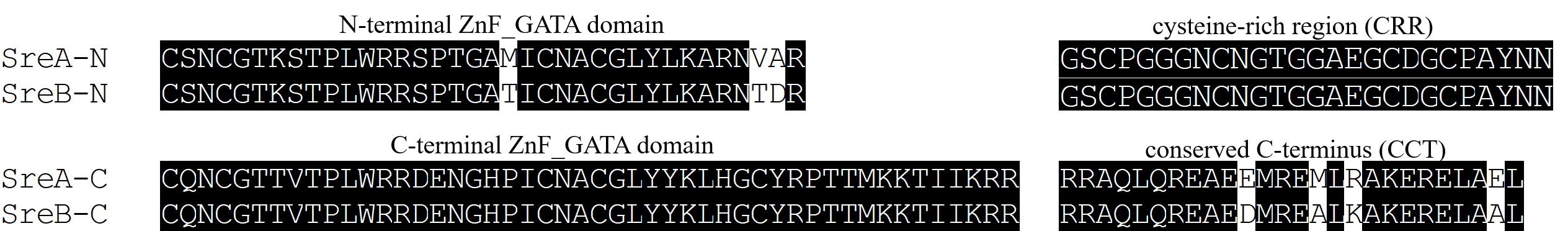

Supplement: Supplementary file 6 — Additional file 6: Figure S3. Alignment of the predicted amino acid sequence of AoSreA with SreB. AoSreA and SreB contained several conserved domains including two ZnF_GATA (N-terminal and C-terminal) separated by a cysteine-rich region (CRR) and a conserved C-terminus (CCT) with a predicted coiled-coil domain. [file 13568_2021_1212_MOESM6_ESM.tif]
